# Additional B‐Lymphocyte Stimulator Inhibitors Treatment Facilitated Glucocorticoid Withdrawal in Lupus Nephritis Patients: A Real‐World Retrospective Observational Single‐Center Cohort Study

**DOI:** 10.1002/mco2.71113

**Published:** 2026-10-29

**Authors:** Jingjing Jin, Meng Tan, Minghui Zhao, Ying Tan

**Affiliations:** ^1^ Renal Division Department of Medicine Institute of Nephrology Key Laboratory of Renal Disease Key Laboratory of Chronic Kidney Disease Prevention and Treatment Peking University First Hospital, Peking University Beijing China; ^2^ Ministry of Health of China, Ministry of Education of China, Research Units of Diagnosis and Treatment of Immune‐Mediated Kidney Diseases, Chinese Academy of Medical Sciences Beijing China; ^3^ Departments of Nephrology Hebei Key Laboratory of Vascular Calcification in Kidney Disease The Fourth Hospital of Hebei Medical University Shijiazhuang Hebei China; ^4^ Hebei Clinical Research Center for Chronic Kidney Disease Shijiazhuang Hebei China; ^5^ Department of Nephrology Peking University First Hospital Taiyuan Branch (Taiyuan Central Hospital of Shanxi Medical University) Taiyuan China

**Keywords:** lupus nephritis, B‐lymphocytestimulator inhibitors, belimumab, glucocorticoids withdrawal, telitacicept

## Abstract

Glucocorticoid (GC) withdrawal remains a major challenge for lupus nephritis (LN). In this 20‐year retrospective cohort, we evaluated whether B‐lymphocyte stimulator (BLyS) inhibitors facilitated GC discontinuation. Among 702 LN patients, 553 were treated with standard‐of‐care alone, and 149 received add‐on BLyS inhibitors (belimumab or telitacicept). Patients receiving BLyS inhibitors achieved a higher rate of GC withdrawal than those receiving standard‐of‐care alone (10.7% vs. 5.61%, *p* = 0.026). Kaplan–Meier analysis confirmed a higher cumulative incidence of GC withdrawal in the BLyS inhibitor group (*p* = 0.001). After adjusting for age, sex, Systemic Lupus Erythematosus Disease Activity Index, acute kidney injury, nephrotic syndrome, and renal histopathology, time‐dependent Cox regression revealed that BLyS inhibitor therapy was independently associated with a higher GC withdrawal rate (hazard ratio [HR] = 7.97, 95% confidence interval [CI]: 4.11–15.5, *p* < 0.001). Exploratory subgroup analyses indicated that among GC‑discontinued patients, Class IV LN predominated in the BLyS inhibitor group, whereas Class V LN was more prevalent in controls. During follow‐up, no renal flares occurred in BLyS inhibitor‐treated patients who discontinued GCs, though there was no significant between‐group difference. In conclusion, add‐on BLyS inhibitor therapy may serve as a potential steroid withdrawal‐permissive approach for LN, pending further prospective validation of its clinical applicability.

## Graphical abstract

Glucocorticoid (GC) withdrawal remains a major challenge for lupus nephritis (LN). Using a 20‐year retrospective cohort, we aimed to evaluate the effect of B‐lymphocyte stimulator (BLyS) inhibitors on successful GC withdrawal in LN patients. Our findings showed that BLyS inhibitor exposure was independently associated with a higher likelihood of GC withdrawal, suggesting a potential steroid withdrawal‐permissive approach in LN patients.

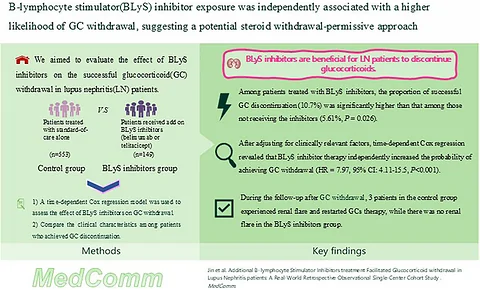

## 1 Introduction

Systemic lupus erythematosus (SLE) is a chronic autoimmune disorder characterized by immune‐mediated multi‐organ involvement [1]. Lupus nephritis (LN) is prevalent in SLE, directly driving long‐term patient morbidity and mortality [2]. Although novel immunosuppressive agents like mycophenolate mofetil (MMF) [3, 4, 5] and voclosporin [6, 7] have improved renal responses, oral glucocorticoids (GCs) remain indispensable during induction and maintenance regimens [8, 9]. Nevertheless, chronic exposure to even low‐dose maintenance steroids (≤5 mg/day) is associated with cumulative risks, such as infections and cardiovascular events [10, 11, 12], ultimately compromising long‐term survival and quality of life. Thus, minimizing or eliminating long‐term steroid toxicity through complete GC withdrawal has become critical.

However, unlike in non‐renal SLE, safe steroid cessation is particularly difficult to achieve in lupus nephritis [13]. Although the 2024 Kidney Disease: Improving Global Outcomes (KDIGO) guidelines suggest considering GC withdrawal in patients with sustained complete renal remission for over 12 months, no standardized withdrawal protocols exist [14]. Furthermore, renal involvement itself confers a high intrinsic risk of relapse upon steroid tapering [15 ]. Therefore, identifying strategies that allow safe GC withdrawal remains an unmet clinical need. The recent emergence of biological agents offers a potential approach to addressing this longstanding challenge.

Aberrant activation of B lymphocytes serves as a core driver in the pathogenesis of SLE [16]. B‐lymphocyte stimulator (BLyS) is a key molecule in the B‐cell activation pathway, regulating the proliferation, differentiation, and maturation of B cells. Studies have demonstrated that BLyS levels are elevated in patients with SLE, and its expression positively correlates with clinical manifestations [17]. Accordingly, BLyS has become a critical therapeutic target for SLE and LN. To date, two BLyS‐targeted biologics have been approved for the treatment of LN. Belimumab is a monoclonal antibody that binds to and neutralizes BLyS [18]. Telitacicept, a fusion protein composed of the IgG Fc fragment and the extracellular domain of the TACI receptor, targets both BLyS and a proliferation‐inducing ligand to prevent them from binding to multiple B‐cell receptors [19]. Clinical trials and real‐world cohort studies have confirmed that add‐on therapy ofbelimumab or telitacicept to standard therapy lowers relapse frequency, accelerates renal responses, and reduces cumulative GC exposure [20, 21, 22, 23, 24, 25]. However, whether BLyS inhibitors effectively facilitate complete, sustained GC withdrawal and protect against post‐withdrawal renal relapses remains unverified.

In this study, we analyzed a longitudinal observational cohort of LN patients followed up for over two decades. We aimed to evaluate the association between BLyS inhibitor add‐on therapy and successful complete GC discontinuation using a time‐dependent Cox proportional hazards model, compare clinicopathological characteristics across different GC withdrawal groups, and assess post‐withdrawal flare outcomes.

## 2 Results

### 2.1 Clinical Characteristics of Patients Between the Two Groups

There were 804 lupus nephritis patients in our cohort. Patients without regular follow‐up (*n* = 102) were excluded from this analysis. Ultimately, 702 patients were enrolled in the primary cohort (Figure 1). Within this cohort, 149 patients receiving belimumab or telitacicept comprised the BLyS inhibitor group, and the remaining 553 patients formed the control group. Baseline clinical characteristics of patients in the two groups are presented in Table 1. At disease onset, patients in the control group were significantly older than those receiving BLyS inhibitors (*p* < 0.001). No significant intergroup differences were observed in terms of sex, rates of acute kidney injury (AKI) and nephrotic syndrome (NS), Systemic Lupus Erythematosus Disease Activity Index (SLEDAI) scores, or renal pathological classification. Likewise, the prevalence of thrombotic microangiopathy (TMA) and tubulointerstitial lesions on renal biopsy was similar between the two groups. Median follow‐up duration in the BLyS inhibitor group was significantly shorter than that in the control group (37 [18, 87] months vs. 53 [22, 122] months, *p* = 0.002).

**FIGURE 1:**
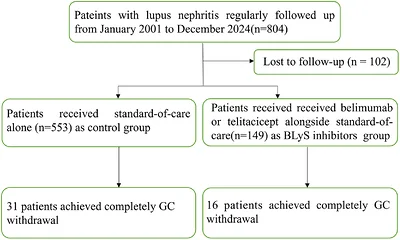
The research flowchart of the study.

**TABLE 1:** Basic characteristics for LN patients between the two groups.

		BLyS inhibitors (*n* = 149)	Controls (*n* = 553)	*p*‐value
Demographics	Age at disease onset, years	28 (20, 36)	33 (25, 45.5)	<0.001
	Female, number (%)	122 (81.9)	445 (80.5)	0.699
Clinical manifestations	SLEDAI at disease onset	16 (12, 20)	15 (10, 18)	0.071
	Acute kidney injury at disease onset, *n* (%)	62 (41.6)	230 (41.6)	0.997
	Nephrotic syndrome at disease onset, *n* (%)	75 (50.3)	312 (56.4)	0.185
	Histopathologic classification, *n* (%)			
	I	0	4 (0.72)	0.584
	II	2 (1.34)	12 (2.17)	0.745
	II + V	0	1 (0.18)	—
	III	11 (7.38)	56 (10.1)	0.312
	III + V	15 (10.1)	48 (8.68)	0.599
	IV	67 (45.0)	228 (41.2)	0.412
	IV + V	27 (18.1)	94 (17.0)	0.747
	V	17 (11.4)	71 (12.8)	0.640
	VI	1 (0.67)	1 (0.18)	0.380
	AI	8 (4, 11)	7 (3, 10.5)	0.179
	CI	1 (0, 3)	2 (0, 3)	0.113
	Others	9 (6.04)	38 (6.87)	0.719

### 2.2 Effect of BLyS Inhibitor Add‐On Therapy on GC Withdrawal

To evaluate the impact of BLyS inhibitor therapy on GC discontinuation among patients with lupus nephritis, we first compared the rates of complete GC withdrawal between the BLyS inhibitor group and the control group during the follow‐up period. In this real‐world cohort, 16 patients (10.7%) in the BLyS inhibitor group achieved GC withdrawal, in contrast to only 31 patients (5.61%) in the control arm. Statistical comparison showed that patients receiving BLyS inhibitors exhibited a significantly higher GC withdrawal rate than those on standard‐of‐care regimens alone (*p* = 0.026). To further characterize the temporal pattern of GC discontinuation, univariable time‐to‐event analysis was performed using the Kaplan–Meier method, with between‐group differences assessed by the log‐rank test. The cumulative incidence of GC withdrawal was markedly elevated in patients with BLyS inhibitor exposure relative to the controls (*p* = 0.001), as visualized in Figure 2. Given that BLyS inhibitors were initiated at variable time points throughout the disease course, a time‐dependent Cox regression model was applied to reduce immortal time bias introduced by conventional statistical approaches. As shown in Table 2, crude univariate analysis revealed that BLyS inhibitor exposure was significantly associated with a higher probability of GC withdrawal (hazard ratio [HR] = 9.09, 95% confidence interval [CI]: 4.92–16.8, *p* < 0.001). After adjustment for basic demographic characteristics, including age and sex, in Model 1, the association remained robust (HR = 9.68, 95% CI: 5.13–18.3, *p* < 0.001). In Model 2, which further adjusted for disease‐specific confounding factors such as SLEDAI score, AKI, NS, and renal histopathological classification, BLyS inhibitor exposure remained independently associated with a higher rate of successful GC withdrawal (HR = 7.97, 95% CI: 4.11–15.5, *p* < 0.001).

**FIGURE 2:**
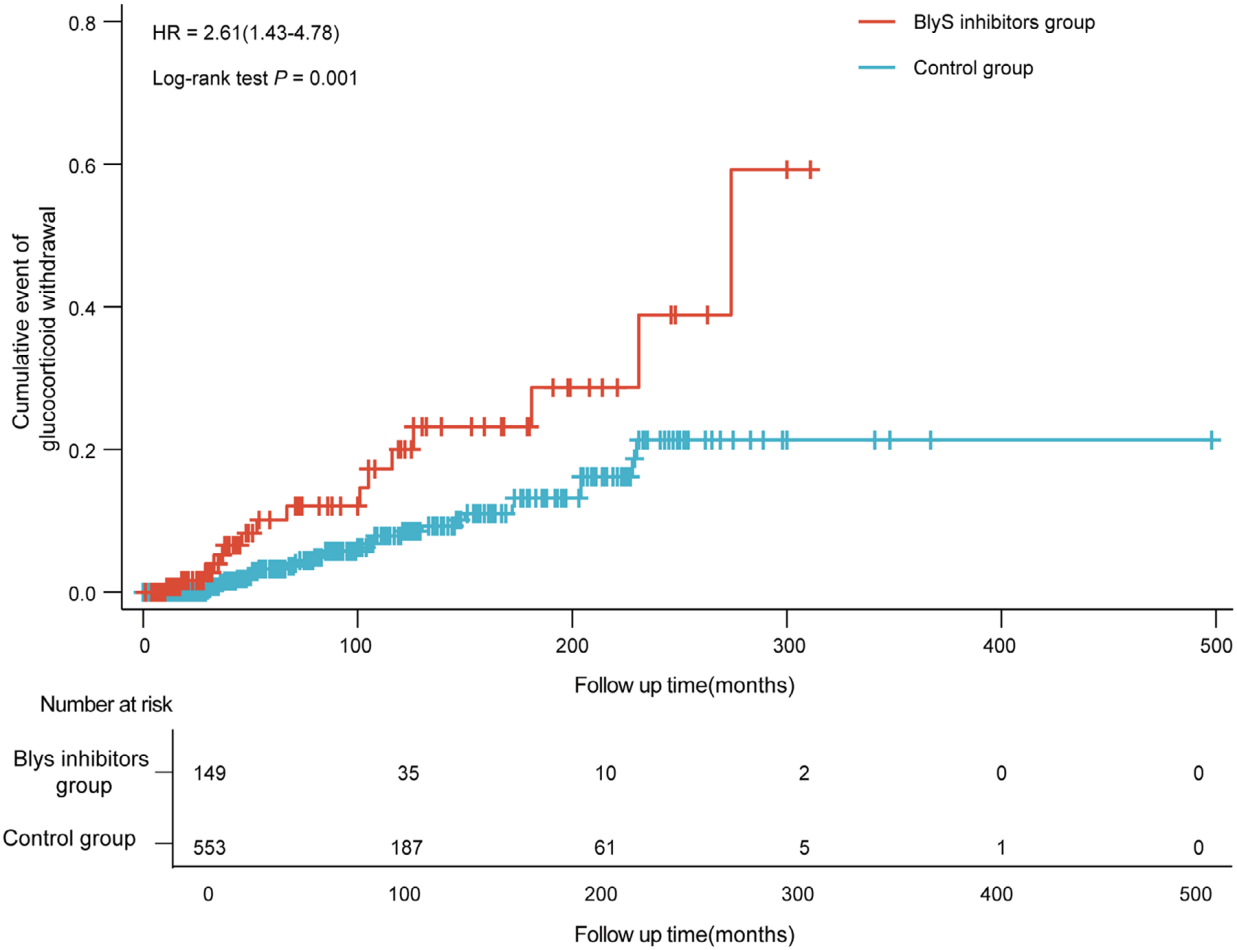
The Kaplan–Meier curve of cumulative events of glucocorticoid withdrawal in the two groups.

**TABLE 2:** The effect of BLyS inhibitors on GC withdrawal by time‐dependent Cox regression analysis.

Characteristic	Crude model HR (95% CI)	*p*‐value	Model 1 HR (95% CI)	*p*‐value	Model 2 HR (95% CI)	*p*‐value
GC withdrawal						
Control	1 (reference)		1 (reference)		1 (reference)	
BLyS inhibitors	9.09 (4.92–16.8)	<0.001	9.68 (5.13–18.3)	<0.001	7.97 (4.11–15.5)	<0.001

*Note*: Crude model: unadjusted. Model 1: adjusted for age and sex. Model 2: further adjusted for SLEDAI, AKI, NS, and renal histopathology in addition to Model 1 variables.

Abbreviations: CI, confidence interval; HR, hazard ratio.

### 2.3 Clinical Characteristics of GC‐Withdrawal Patients Between the Two Groups

We further analyzed the baseline clinical characteristics of patients who successfully achieved GC withdrawal between the two groups. As summarized in Table 3, GC withdrawal patients in the BLyS inhibitor group had higher disease activity at onset, with a mean SLEDAI score of 17.6 ± 4.4 versus 13.5 ± 5.3 in the control group (*p* = 0.011). In terms of renal pathology, the BLyS inhibitor group had a substantially higher proportion of Class IV LN among patients who successfully achieved GC withdrawal (81.3% vs. 38.7%, *p* = 0.006), including two cases with concomitant TMA. In contrast, Class V LN was more prevalent among controls who achieved GC withdrawal (25.8% vs. 0%, *p* = 0.038). These differences indicate that add‐on BLyS inhibitor therapy may improve the likelihood of achieving complete steroid cessation in patients with active and proliferative LN.

**TABLE 3:** Basic characteristics for GC‐withdrawal patients between the two groups.

		BLyS inhibitors (*n* = 16)	Controls (*n* = 31)	*p*‐value
Demographics	Age at disease onset, years	29.2 ± 13.4	33.0 ± 15.9	0.411
	Female, number (%)	12 (75.0)	27 (87.1)	0.416
Clinical manifestations	SLEDAI at disease onset	17.6 ± 4.4	13.5 ± 5.3	0.011
	Acute kidney injury at disease onset, *n* (%)	4 (25.0)	4 (12.9)	0.416
	Histopathologic classification, *n* (%)			
	I	0	1 (3.23)	—
	II	1 (6.25)	1 (3.23)	—
	III	1 (6.25)	2 (6.45)	—
	III + V	1 (6.25)	2 (6.45)	—
	IV	13 (81.3)	12 (38.7)	0.006
	IV + V	0	5 (16.1)	0.150
	V	0	8 (25.8)	0.038

Then we further explored the clinical characteristics of patients at the time of GC withdrawal. The cumulative duration of prior GC exposure was comparable between the BLyS inhibitor group and the control group, with no statistically significant difference (*p* = 0.895). However, 46.7% (7/15) of proliferative LN patients in the BLyS inhibitor group accomplished GC withdrawal within 4 years after disease onset, which was significantly higher than the corresponding rate of 9.52% (2/21) in the control group (*p* = 0.019). Moreover, 19.4% (6/31) of controls retained persistent immunological activity (anti‐dsDNA positivity or hypocomplementemia) at the time of GC cessation, whereas all patients in the BLyS inhibitor group achieved complete serological remission.

In addition to GC exposure duration and serological activity, we also compared the usage of maintenance immunosuppressant regimens between the two groups at the time of GC withdrawal. As illustrated in Figure 3, combined therapy with MMF and hydroxychloroquine (HCQ) was more prevalent in both groups. In the BLyS inhibitor group, 68.8% (11/16) of patients were maintained on MMF plus HCQ, 12.5% (2/16) on cyclosporine (CsA) plus HCQ, 6.25% (1/16) on CsA plus leflunomide (LEF), 6.25% (1/16) on triple therapy consisting of MMF, tacrolimus (Tac), and HCQ, and 6.25% (1/16) on LEF plus HCQ. In the control group, MMF plus HCQ accounted for 54.8% (17/31), LEF plus HCQ for 29.0% (9/31), and CsA plus HCQ for 9.68% (3/31), while a small proportion of patients received azathioprine (AZA) plus HCQ (3.23%, 1/31; only in the control group) or triple therapy consisting of MMF, Tac, and HCQ (3.23%, 1/31). Further chi‐square analysis confirmed that no significant intergroup difference was detected in the overall distribution of immunosuppressive agents at GC withdrawal (all *p* > 0.05).

**FIGURE 3:**
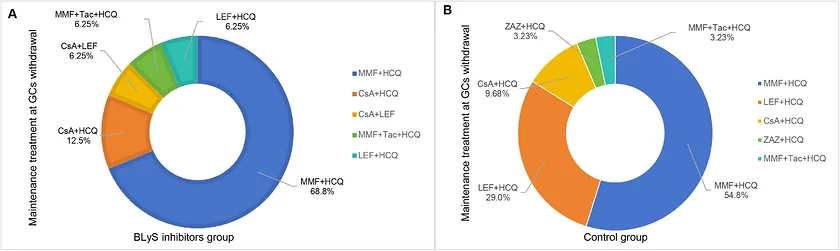
The utilization of other therapeutic agents in the two groups at glucocorticoid withdrawal. Csa, cyclosporin A; HCQ, hydroxychloroquine; LEF, leflunomide; MMF, mycophenolate mofetil; Tac, tacrolimus.

### 2.4 Prognosis After GC Withdrawal Between the Two Groups

Finally, we compared post‐withdrawal renal outcomes between the two groups. Two patients in the control group who received belimumab after GC discontinuation for relapse prevention were excluded from post‐withdrawal outcome analyses. The follow‐up duration after GC cessation in the BLyS inhibitor group was 22.7 ± 13.3 months, compared with 39.8 ± 20.7 months in the controls (*p* = 0.005). In the BLyS inhibitor group, six patients discontinued biologicals within 6 months post‐withdrawal, seven extended the dosing intervals to every 2–3 months, and three maintained standard monthly therapy. During post‐withdrawal monitoring, no renal flares occurred in the BLyS inhibitor group. In comparison, three patients (9.68%) in the control group suffered from renal flares requiring steroid re‐initiation. Although the Kaplan–Meier curves showed a trend toward improved renal flare‐free survival in the BLyS group, the difference did not reach statistical significance by the log‐rank test (*p* = 0.344, Figure 4).

**FIGURE 4:**
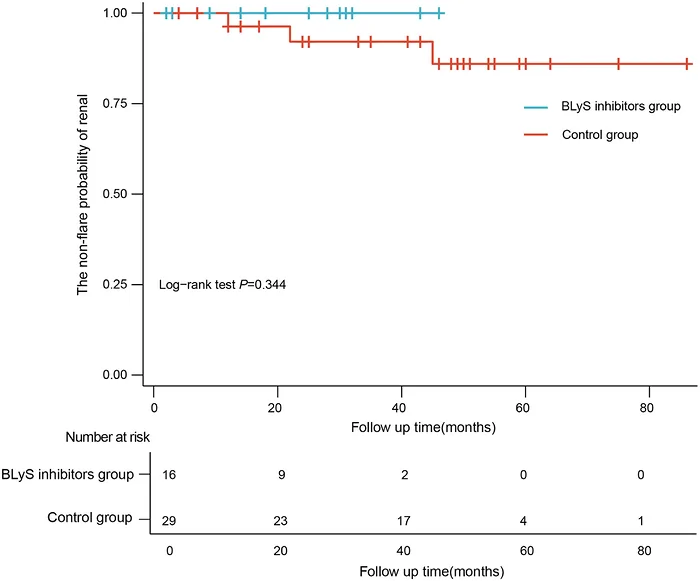
The Kaplan–Meier curve of renal flare in the two groups after glucocorticoid withdrawal.

## 3 Discussion

This retrospective comparative study extends current evidence regarding BLyS inhibitors for complete GC discontinuation in lupus nephritis. In this study, we enrolled a large cohort and stratified patients according to whether they received BLyS inhibitors. Multivariable adjusted analysis demonstrated that add‐on BLyS inhibitor therapy was independently associated with the likelihood of successful glucocorticoid withdrawal. Further exploratory subgroup analyses suggested that BLyS inhibitors were associated with GC discontinuation in patients with proliferative LN. During follow‐up after GC withdrawal, no renal flares were observed in the BLyS inhibitor group, whereas a renal flare rate of 9.68% was observed in the control group. Our study suggests that add‐on BLyS inhibitor therapy increases the probability of achieving successful GC withdrawal.

To our knowledge, this is the first large cohort study to specifically evaluate complete GC withdrawal in BLyS inhibitor‐treated patients with lupus nephritis. BLyS inhibitor exposure remained strongly associated with successful GC withdrawal after multivariable adjustment. Although glucocorticoids remain a cornerstone for the treatment of LN, their associated risks, particularly with long‐term exposure, have been well established [26]. Therefore, GC tapering and withdrawal constitute one of the core goals in LN management. The 2025 Chinese guidelines recommend considering GC withdrawal in patients sustaining a complete renal response for over 12 months on maintenance therapy [27]. However, evidence supporting the optimal timing and implementation of complete GC withdrawal remains limited. In recent years, the approval of belimumab and telitacicept for SLE treatment has attracted widespread attention from clinicians and patients. Numerous randomized controlled trials and real‐world studies have confirmed the GC‐reducing effect of belimumab and telitacicept. The BLISS‐52 trial showed that belimumab significantly increased the proportion of patients achieving a 50% reduction in glucocorticoid exposure. The post hoc pooled analysis of BLISS‐52 and BLISS‐76 indicated that the belimumab group had more patients with GC dose reduction and fewer with dose escalation versus controls, with substantially lower overall glucocorticoid use [28]. Similarly, the Phase 3 trial demonstrated that telitacicept enabled 44.9% of its participants to meet the glucocorticoid tapering target (prednisone equivalent ≤7.5 mg/day or ≥25% reduction from baseline) at Weeks 44–52, compared with 34.7% for placebo [19]. Our previous real‐world evidence also revealed that add‐on telitacicept significantly reduced daily steroid dosage at the last follow‐up [20]. However, evidence regarding the role of these two BLyS inhibitors in glucocorticoid discontinuation remains limited. Only data from case reports have suggested the plausible clinical utility of belimumab for facilitating early GC withdrawal among patients with lupus‐associated thrombocytopenia [29] and renal transplant recipients with LN [30]. This study extends prior evidence on the established steroid‐sparing effects of BLyS inhibition, expanding its scope from partial GC dose reduction to complete GC discontinuation. However, complete GC withdrawal in LN is unlikely to depend on a single therapeutic agent; rather, it requires sustained renal remission, well‐controlled extrarenal involvement, appropriate background immunosuppression, and careful patient selection. Of note, these findings require further validation in larger, multicenter prospective studies.

Interestingly, successful GC withdrawal was also observed in BLyS inhibitor‐treated patients with proliferative LN and high baseline disease activity. Our prior work [31] has indicated that patients presenting with nephrotic syndrome at disease onset had lower odds of achieving successful GC tapering and discontinuation. In the present study, we found that patients who successfully discontinued GC in the BLyS inhibitor group had higher disease activity and a greater proportion of Class IV LN at disease onset, whereas Class V LN dominated in the control group. This observation suggests that severe initial presentation does not necessarily preclude subsequent GC withdrawal once sustained renal control has been achieved, which is also supported by external evidence. A post hoc analysis of proliferative LN patients receiving MMF standard therapy in the BLISS‐LN trial [32] confirmed greater belimumab‐derived benefits in this subgroup: add‐on belimumab significantly improved renal responses and preserved renal function relative to placebo, with more prominent renal benefits than those seen in the overall BLISS‐LN population, alongside a favorable safety profile and fewer serious adverse events. Likewise, a multicenter Spanish cohort study demonstrated that early initiation of belimumab‐based therapy for proliferative LN achieved high renal remission rates and facilitated marked GC tapering [33]. Extending beyond belimumab, a retrospective proliferative LN cohort study further demonstrated that telitacicept yielded comparable clinical efficacy and safety profiles relative to belimumab, facilitated GC and immunosuppressant dose reduction, and had the potential for early remission induction [34]. In all, these studies suggest that both belimumab and telitacicept may permit substantial GC reduction in proliferative LN. No renal flares occurred among patients in the BLyS inhibitor group after GC withdrawal in this study, whereas three patients in the control group experienced a recurrence of kidney disease.

In clinical practice, nearly a quarter of patients with lupus nephritis suffer a disease relapse within 3–4 years after achieving remission [24], and the risk of recurrence may be further elevated following complete glucocorticoid withdrawal. LN flares cause irreversible nephron loss and markedly shorten the effective functional lifespan of the kidney. Recurrent episodes of LN may ultimately lead to renal failure and raise the risk of cardiovascular disease [35]. Thus, effective prevention of LN flares constitutes a core therapeutic goal for the long‐term management of lupus nephritis. The secondary analysis of the BLISS‐LN trial set Week 24 as the observational starting point, at which all patients had completed induction therapy and their glucocorticoid dosage was tapered to ≤10 mg/day, to explore the protective effects of belimumab on LN flares and renal function. The results confirmed that combination therapy with belimumab could effectively delay renal function deterioration and reduce the risk of overt LN flares, regardless of patients’ background treatment regimens, renal histopathological classifications, and baseline proteinuria levels [24]. In the present study, 62.5% of patients in the BLyS inhibitor group remained on continuous BLyS inhibitor maintenance therapy during the post‐GC withdrawal follow‐up period. Therefore, the present data cannot distinguish whether the low flare rate is attributed to prior BLyS inhibitor exposure, ongoing BLyS inhibitor maintenance, rigorous patient selection, or the relatively shorter post‐withdrawal follow‐up duration. Nevertheless, these findings suggest that the addition of BLyS inhibitors holds potential to reduce the risk of disease recurrence among LN patients following GC withdrawal, which remains to be confirmed by long‐term prospective cohorts with extended post‐withdrawal follow‐up.

However, the study is limited by its single‐center retrospective design. Moreover, although time‐dependent modeling and multivariable adjustment were applied, residual confounding due to treatment selection and unmeasured clinical factors cannot be completely excluded because of the retrospective observational design. Finally, the relatively small number of patients achieving GC withdrawal limited statistical power for secondary analyses and precluded detailed evaluation of treatment heterogeneity among different LN subtypes.

In conclusion, BLyS inhibitor exposure was independently associated with a higher likelihood of successful complete GC withdrawal in this real‐world LN cohort. Our findings indicate that add‐on BLyS inhibitor therapy may serve as a promising steroid withdrawal‐permissive strategy for patients with lupus nephritis, facilitating successful GC discontinuation without an apparent increase in renal flares during the observed follow‐up period. Nevertheless, these results should be interpreted with caution given the inherent limitations of this single‐center retrospective study, and larger prospective cohorts with extended post‐GC‐withdrawal follow‐up are warranted to validate the long‐term efficacy and safety of this therapeutic strategy.

## 4 Materials and Methods

### 4.1 Study Design and Participants

In strict compliance with the Strengthening the Reporting of Observational Studies in Epidemiology (STROBE) reporting guidelines [36], this retrospective cohort study analyzed 804 consecutive patients with lupus nephritis admitted to Peking University First Hospital between January 2001 and December 2024. Inclusion criteria were: (i) age ≥18 years; (ii) fulfillment of American College of Rheumatology 1997 revised lupus nephritis criteria; and (iii) regular outpatient follow‐up. Patients lacking consistent follow‐up (*n* = 102) were excluded, leaving 702 patients in the primary cohort (Figure 1). Ethical approval was granted by the institutional review board (Approval No. 2017[1333]), and written informed consent was obtained from all patients.

### 4.2 Treatment Regimens

All patients received standard‐of‐care induction/maintenance therapy based on KDIGO guidelines. BLyS inhibitors were initiated according to physician judgment based on clinical indications, including persistent renal or systemic disease activity despite standard therapy, difficulty achieving glucocorticoid tapering, recurrent disease activity, persistent serological abnormalities, or the need for steroid‐sparing treatment. Patients in the BLyS inhibitor group received belimumab (10 mg/kg intravenously on Days 1, 15, 29, then every 4 weeks) or telitacicept (80/160 mg subcutaneously weekly) added to standard care. Controls received standard therapy alone.

### 4.3 Data Collection

Study participants underwent regular follow‐up at the lupus nephritis outpatient clinic. Detailed clinical data were collected, including the age, clinical manifestations, serum white blood cell count, hemoglobin level, platelet count, serum complement component 3 (C3) and component 4 (C4) levels, anti‐double‐stranded DNA (ds‐DNA) antibody level, serum creatinine level, renal histopathological data, and therapies at the onset of LN. Information also covered the time of glucocorticoid discontinuation, the characteristics and treatment at the time of discontinuation, and the occurrence of disease flare‐ups after glucocorticoid withdrawal.

### 4.4 Definitions and Outcomes

GC discontinuation was defined as total discontinuation of systemic steroids. The SLEDAI was used to assess disease activity. The renal flare was defined by the following manifestations that required reinitiation of glucocorticoid therapy: a recent 50% rise in serum creatinine alongside active urinary sediments, newly emerging nephrotic syndrome, or proteinuria above 1.5 g/day without other etiologies in patients previously free of proteinuria [20].

### 4.5 Statistical Analysis

SPSS version 19.0 and R version 4.5.2 were used for all data analyses and plotting. The R packages involved include readxl, survival, survminer, ggplot2, dplyr, and openxlsx. Normally distributed continuous variables were reported as mean ± standard deviation (SD), and non‐normally distributed continuous variables as median (25th, 75th percentile). Categorical data were summarized by frequencies and percentages. Intergroup differences were assessed via *t*‐tests, Mann–Whitney *U* tests, or chi‐square (*χ* ^2^) tests. Kaplan–Meier curves and multivariable time‐dependent Cox regression analysis were used to analyze patient prognosis and mitigate potential bias. Statistical significance was defined as a two‐tailed *p*‐value less than 0.05.

## Author Contributions

Ying Tan and Minghui Zhao designed this study; Jingjing Jin and Meng Tan carried out this study and wrote the draft; Ying Tan revised this paper. All authors have read and approved the final manuscript.

## Funding

This work was supported by grants from the Beijing Natural Science Foundation (7242143); Capital's Funds for Health Improvement and Research (2024‐2‐4076); Taiyuan Bureau of Science and Technology, Science, Technology, and Innovation Program of National Regional Medical Center (202225); National High Level Hospital Clinical Research Funding (Interdisciplinary Research Project of Peking University First Hospital) (2024IR42); the CAMS Innovation Fund for Medical Sciences (2019‐I2M‐5‐046); Peking University First Hospital Cross‐Research Project (No. 2023IR35); National Key Research and Development Program of China (2023YFC3503504); and Medical Science Research Project of Hebei (20240407).

## Ethics Statement

The study was conducted in accordance with the Declaration of Helsinki and approved by the Local Ethical Committees of Peking University First Hospital (No. 2017 [1333]). Written informed consent was obtained from all of the participants.

## Conflicts of Interest

The authors declare no conflicts of interest.

## Data Availability

All data generated or analyzed during this study are included in this article. Further inquiries can be directed to the corresponding author.
